# Measuring the outcomes of long-term care for unpaid carers: comparing the ASCOT-Carer, Carer Experience Scale and EQ-5D-3 L

**DOI:** 10.1186/s12955-019-1254-2

**Published:** 2019-12-16

**Authors:** Stacey Rand, Juliette Malley, Florin Vadean, Julien Forder

**Affiliations:** 1 Personal Social Services Research Unit (PSSRU), Cornwallis Building, University of Kent, Canterbury, CT2 7NF UK; 2 The Care Policy and Evaluation Centre (CPEC), London School of Economics and Political Science, Houghton Street, London, WC2A 2AE UK

**Keywords:** Quality of life, Caregiver, Social care, Long-term care, ASCOT-Carer

## Abstract

**Background:**

The ASCOT-Carer and Carer Experience Scale are instruments designed to capture aspects of quality of life ‘beyond health’ for family carers. The aim of this study was to compare and validate these two carer care-related measures, with a secondary aim to compare both instruments to the three-level EQ-5D (EQ-5D-3 L) measure of health-related quality of life.

**Methods:**

An interview survey was conducted with 387 carers of adults who used long-term care (also known as social care) support in England. Construct validity by hypothesis testing was assessed using Pearson correlation coefficient. Exploratory factor analysis was also applied to investigate the dimensionality of the combined items from the ASCOT-Carer and CES (as measures of carer quality of life ‘beyond health’) and the EQ-5D (as a measure of health-related quality of life).

**Results:**

In the construct validity analysis, hypothesised differences in correlations were observed with two exceptions. The exploratory factor analysis indicated that the ASCOT-Carer, CES and EQ-5D-3 L items loaded onto three separate factors. The first factor comprised the seven ASCOT-Carer items plus two CES items (activities outside caring, support from friends and family). The second factor comprised three of the six CES items (fulfilment from caring, control over caring and getting on with the person you care for). The third factor included four of the five EQ-5D-3 L items.

**Conclusion:**

The findings indicate that the ASCOT-Carer, CES and EQ-5D-3 L capture separate constructs of social care-related quality of life (ASCOT-Carer) and carer experience (CES), which partially overlap in relation to activities outside caring and social support, and health-related quality of life (EQ-5D-3 L). The ASCOT-Carer and CES are both promising measures for the evaluation of social care support for carers that capture aspects of quality of life ‘beyond health’. The choice of whether to use the ASCOT-Carer or CES depends on the study objectives.

## Introduction

Social care (or long-term care) refers to services that seek to maintain or improve quality of life of people who experience difficulties with everyday activities due to long-term health conditions, disability, or age-related impairments (for example, home care) [1, 2]. More recently, the scope of social care policy and interventions have broadened to consider family or friends who support adults with care needs (for example, carers’ support groups or support to stay in employment) [3]. These developments recognise the significant contribution of informal care to long-term care systems [4, 5] and also the needs of carers in terms of the impact of caregiving on their own health, wellbeing and ability to remain in employment or education [6–9].

In England, there has been a trend towards recognising carers as users of social care services in their own right [8, 9]. The Care Act (2014) places responsibility on local authorities to identify and address the needs of carers alongside those of the adults they support. Therefore, especially in the context of outcomes-based management and administration in public services [10], there is policy-driven interest in considering the quality of life outcomes of informal carers alongside adults with care needs [11, 12]. There are also other arguments for the measurement of carers’ outcomes alongside those of the patient or service user. If the aim of publicly-funded health and social care services is to maximise outcomes through resource allocation on a societal-level, for example, it has also been argued that the impact on carers should also be considered [13–17]. In addition, the benefits of an intervention may be over- or underestimated if its effects on informal carers’ quality of life are not also considered.

In the area of healthcare, the benefits of policy or interventions may be calculated using quality-adjusted life years (QALY), which is the product of life expectancy and health-status of an individual during those years. In calculating QALYs, the EuroQol five-dimension questionnaire (EQ-5D), a measure of health-related quality of life, is widely used [18]. The EQ-5D-3 L captures individual functioning in five health-related domains: pain, mobility, usual activities, anxiety/depression, and self-care [19, 20]. The responses to the five questions, from no problems (1) to severe problems (3), are combined into an EQ-5D health state, for example, 1–1–2-1-3. Using weights developed from preference studies, these states may be converted into a single summary index value, from 0 (being dead) to 1 (in perfect health) [21]. In the evaluation of the (cost-)effectiveness of interventions designed to support carers, however, the attributes captured by the EQ-5D or other health-related quality of life (QoL) instruments may not relate to carers’ concerns or the broader aspects of QoL that may be affected by health and social care interventions [16]. Furthermore, as EQ-5D has been found to lack specificity and sensitivity in the evaluation of *social care* interventions for service users [22], the impact of social care interventions or policy for carers may likewise be captured more effectively by measures of broader QoL (‘beyond health’): for example, social relationships, support and connectedness, autonomy and control or feeling supported and encouraged in the caring role [16, 23–28].

The ASCOT-Carer four-level interview (INT4) [25, 26] and Carer Experience Scale (CES) [27–29] are instruments designed for economic evaluation of the impact of services on carers beyond health. The ASCOT-Carer is part of the Adult Social Care Outcomes Toolkit (ASCOT), which is a suite of measures suitable for the economic evaluation of social care interventions or policy [1, 30–35]. The measures capture the social care-related quality of life (SCRQoL) of carers and users of adult social care services [25, 26], which relates to aspects of quality of life that may be influenced by social care services [22, 30]. In the UK, social care refers to a range of long-term care services from residential care through to community-based services for adults with care needs that may indirectly also support carers (e.g. domiciliary care, equipment and home adaptations), as well as services directed at the specific needs of carers (e.g. support groups, information and advice) [24, 36].

The seven ASCOT-Carer social care-related quality of life attributes (see Table 1) were identified through focus groups with care managers and carers [37], one-to-one cognitive interviews with carers [38], and a pilot survey of carers in England [39]. Based on this preliminary work, a three-level response version with seven items was developed, the Carer SCRQoL [38, 39]. The Adult Social Care Outcomes Framework (ASCOF) in England includes an abbreviated version of this instrument with only six attributes. (*Time and space to be myself* is omitted.) This is used as an overarching indicator of carer-reported QoL (ASCOF 1D) [12]. Further research refined the questionnaire to expand the number of response options from three to four-levels [26], which correspond to the ASCOT outcomes states of ideal state, no needs, some needs and high-level needs [30]. The questionnaire was also harmonised with the user version of ASCOT and adapted to incorporate feedback from carers in cognitive debriefing interviews [30].

**Table 1:** Description of ASCOT-Carer and Carer Experience Scale attributes

ASCOT-Carer Attribute	Description
Occupation	Being able to do the things you value and enjoy, whether it be paid or unpaid work, caring for others, or leisure activities
Control over daily life	Being able to choose what to do and when to do it; having control over daily activities
Self-care	Feeling able to look after yourself as well as you want to: for example, eating well, getting enough sleep
Personal safety	Feeling safe and secure, where concerns about safety may include fear of abuse, physical harm or accidents that arise as a result of caring
Social participation and involvement	Being able to sustain the relationships with friends and family, and feeling involved or part of a community, as much as you want to
Space and time to be yourself	Having the space and time you want away from the caring role and the responsibility of caregiving
Feeling encouraged and supported	Feeling encouraged and supported by professionals, care workers and others, in your role as a carer
Carer Experience Scale Attribute	Description
Activities outside of caring	Being able to do a range of things you want outside of caring (e.g. socialising, physical activity and spending time on hobbies, leisure or study)
Support from family and friends	Amount of personal help in caring and/or emotional support from family, friends, neighbours or work colleagues
Assistance from organisations and government	Amount of help from public, private or voluntary groups in terms of benefits, respite and practical information
Fulfilment from caring	Frequency of experiencing positive feelings from providing care, which may come from: making the person you care for happy, maintaining their dignity, being appreciated, fulfilling your responsibility, gaining new skills or contributing to the care of the person you look after
Control over caring	Being able to influence the overall care of the person you look after
Getting on with the person you care for	Frequency of being able to talk with the person you look after and discuss things without arguing

The Carer Experience Scale is designed to capture the effect of health and social care interventions on aspects of carers’ experience ‘beyond health’ in economic evaluation [27–29]. It is a measure of carers’ experiences related to the *process* of providing care [27, 29]. The six conceptual attributes considered in the scale were developed through a meta-ethnography to synthesise the findings of qualitative research on caregiving experience followed by semi-structured interviews with carers to establish the content validity of the identified attributes [27]. The final set of six attributes are activities outside of caring, support from friends and family, assistance from government and organisations, fulfilment from caring, control over caring and getting on with the care recipient (see Table 1). The three levels of response to each attribute either relate to amount (‘a lot’, ‘some’, ‘a little’) or frequency (‘rarely’, ‘sometimes’, ‘mostly’) [27]. Unlike the CES, the ASCOT-Carer quality of life attributes relate to social care outcomes rather than caregiving experience: for example, the ASCOT-Carer construct of control captures whether the carer feels that s/he has sufficient control over their daily life and activities. By contrast, the CES item on control relates more narrowly to the carer’s control over aspects of caregiving only (see Table 1).

This article presents a comparison of the ASCOT-Carer and CES, as two measures of carer outcomes ‘beyond health’. This is to understand the overlap and distinctiveness of the constructs captured by the two measures, so they may be used appropriately. Both measures were also compared to the three-level EQ-5D (EQ-5D-3 L), to establish whether they measure the construct of carer outcomes ‘beyond health’. A secondary aim was to contribute to the evidence of the construct validity of the ASCOT-Carer [25] and CES [29] by hypothesis testing.

## Method

### Design and participants

The study sample comprises carers who participated in the *Identifying the Impact of Adult Social Care* study, which is described in more detail elsewhere [23, 25]. Carers were recruited in 22 English local authorities. All carers supported someone who used publicly-funded adult social care services, who received support due to physical or intellectual disabilities or mental health conditions, and had taken part in an interview for the *Identifying the Impact of Adult Social Care* study.

In the interview with social care service recipients, participants were asked whether they had been helped by family and friends with regard to activities of daily living (ADLs) and instrumental activities of daily living (IADLs), as well as the number of hours of help received in the last week. At the end of the interview, all participants who had reported that they had received help was asked whether they agreed for their primary carer (defined as the person who had provided the most hours of care in the past week) to be invited to also take part in an interview. A total of 990 interviews with social care recipients were conducted. From these, 739 carers were identified; 510 (69%) were invited to take part in an interview. A total of 387 interviews with carers were completed, either face-to-face (*n* = 336) or telephone (*n* = 51), between June 2013 and March 2014.

Ethical approval for the study was obtained from the national social care research ethics committee in England (12/IEC08/0049).

### Quality of life measures

Three quality of life measures are compared in this study: a measure of health-related quality of life (the EQ-5D-3 L) and two measures of the QoL ‘beyond health’ (the ASCOT-Carer and CES).

### ASCOT-Carer

The Adult Social Care Outcomes Toolkit for Carers (ASCOT-Carer) is a measure of social care-related quality of life [25, 26]. The ASCOT-Carer interview (INT4) used in this study captures three measures of SCRQoL: (1) current SCRQoL; (2) expected SCRQoL; and (3) SCRQoL ‘gain’, an estimate of the impact of social care on QoL using counterfactual self-estimation methodology developed as part of the ASCOT for service users [30, 33] and applied to the carer version of the instrument [26]. In this paper, we consider only current social care-related quality of life. An index score of zero (worst possible SCRQoL) to one (best possible SCRQoL) is calculated by the sum of preference-weights for each selected outcome level, which were developed through best-worst scaling with a general population sample in England [40].

### Carer experience scale

The Carer Experience Scale is a measure of caregiving experience [27–29]. A total index score is calculated by summing the preference-weights that correspond to the level selected for each domain. The preference weights were developed through best-worst scaling in a sample of carers in the United Kingdom [29]. The index score ranges from 0 to 100, where zero represents the lowest caregiving experience state and 100 the highest caregiving experience state.

### EQ-5D-3 l

The EQ-5D-3 L is a five-item instrument that measures health-related quality of life (HRQoL) [19, 20]. The items capture five HRQoL attributes: mobility; self-care; usual activities; pain and discomfort; and anxiety/depression. Each item has three levels of response: no, some or extreme problems. Health states are converted into an index score by calculating the sum of preference-based weights, which correspond to the selected level for each item. The index score range from − 0.594 to 1, where − 0.594 represents extreme problems in all five attributes and 1 represents full health. The preference-weights used to determine the index score were calculated from a study of adults in the United Kingdom [21].

### Other measures

The questionnaire also included items to capture the sample characteristics, including the sex, age and employment status of the carers, as well as the context of care (i.e. whether the carer and care-recipient live together, the duration and intensity of caregiving) using items adapted from the household survey of carers in England 2009/10 [41]. The functional ability of the care-recipient was collected through the care-recipient interview, as a self-report of whether s/he found it difficult to complete a list of eight activities of daily living or instrumental activities of daily living (I/ADLs), e.g. getting washed, dressed, in/out of bed. These ratings were combined into a score from none (0) to all eight I/ADLs (8).

The carer questionnaire also included items or scales for construct validity testing by hypothesis testings (see Table 4). These include the perceived choice subscale of the self-determination scale (SDS) [42], which reflects the extent to which individuals feel that they have a choice with respect to their behaviour. The subscale captures the subjective degree of perceived autonomy in everyday activities. Five items are rated from 1 to 5, where a higher rating represents a higher sense of autonomy. The overall subscale score is the average score across the five items.

Positive aspects of the relationship between the carer and care-recipient was measured using the relationship rewards scale, which includes four items: feeling happy with the relationship; the relationship making the carer feel good about themselves; feeling emotionally close to the care recipient; and feeling bored in the relationship [43]. The frequency of these experiences was rated from never (0) to always (3). The overall score was the sum of the four item scores, with the fourth item reverse scored, to form a scale from 0 (lowest relationship reward) to 12 (highest relationship reward). Social loneliness was measured by the three-item UCLA loneliness subscale [44]. The sum score of the items forms a scale from 3 to 9. Higher scores represent a higher degree of perceived loneliness. Frequency of contact with friends and family by telephone or face-to-face was rated on five-point scales from ‘never’ (1) to ‘on most days’ (5).

The questionnaire also included a self-rated health question to rate current health on a five-point scale from very bad (1) to very good (5). Overall quality of life was rated on a seven-point scale from ‘so bad it couldn’t be worse’ (1) to ‘so good it couldn’t be better’ (7). The carers’ satisfaction with social care support (i.e. all social care services used by the carer and/or care recipient) was rated on the seven-point scale from extremely dissatisfied (1) to extremely satisfied (7).

### Analysis

Analyses were conducted in Stata version 13 [45].

### Construct validity

The construct validity of the EQ-5D-3 L, CES index, ASCOT-Carer SCRQoL index scores was assessed by testing a priori hypotheses of the relationship between measures and other items or scales, which were developed through literature review, previous research and discussion within the research team. The hypothesised correlations were evaluated by Pearson correlation coefficients. The expected associations were considered to be significant where the difference between the Pearson correlation coefficients were greater than 0.1, which has been applied as the smallest expected difference between correlation coefficients in other construct validity studies [46, 47].

The hypothesised associations are outlined in Table 4. First, we anticipated that ASCOT-Carer and CES would be more strongly correlated to each other than either scale with EQ-5D-3 L because the ASCOT-Carer and CES intend to measure broader aspects of QoL than health-related QoL (1). We also expected that the ASCOT-Carer would be less strongly correlated than the EQ-5D-3 L to self-rated health (2). Likewise, we expected both the ASCOT-Carer SCRQoL and CES to be more strongly correlated to each other than the EQ-5D-3 L on these indicators of health (14).

It was also anticipated that the ASCOT-Carer and CES would be more strongly correlated to overall quality of life rated on a 7-point Likert scale than the EQ-5D-3 L, which focuses more narrowly on health-related aspects of QoL, and the CES (3, 14). The ASCOT-Carer was expected to be more strongly correlated to the perception of ability to make choices on the SDS choice subscale than the EQ-5D-3 L, which does not include choice or control as a domain, or the CES, which only captures choice in relation to the caregiving role rather than more broadly (4, 10). Relationship rewards, or positive aspects of the relationship between the carer and care recipient, were expected to be less strongly associated with EQ-5D-3 L than ASCOT-Carer (5), but more strongly associated to the CES than ASCOT-Carer since one of the five CES domains captures how well the carer gets on with the care recipient (11*)*.

It was expected that the UCLA loneliness subscale and frequency of contact with friends or family would be more strongly related to ASCOT-Carer than EQ-5D-3 L since social loneliness forms part of the SCRQoL construct; however, it is not included in the EQ-5D-3 L construct of HRQoL (6, 7, 8). By contrast, it was anticipated that the ASCOT-Carer would be more strongly correlated to UCLA loneliness score than the CES (12) because the CES captures perceived social support, rather than social loneliness. As such, since frequency of social contact may be similarly associated with the constructs of social loneliness and social support, the correlation between ASCOT-Carer or CES and frequency of contact with friends/family was anticipated to be similar (15, 16).

Finally, it was anticipated that the ASCOT-Carer would be more strongly related than the EQ-5D-3 L to satisfaction with social care services (9), while there would be correlation of approximately equal strength for the ASCOT-Carer and CES (17) since both instruments capture care-related aspects of quality of life and experience respectively.

### Exploratory factor analysis

Exploratory factor analysis (EFA) is used to identify the underlying dimension(s) within a measurement instrument that may form subscales [48]. In this study, EFA was used to investigate the structural validity (dimensionality) of the ASCOT-Carer, CES and EQ-5D-3 L. An EFA was applied to all items from each of the three measures. A similar method has been used to explore the dimensionality of the corresponding measures of ICEpop CAPability measure for Older people (ICECAP-O) [49, 50] and ASCOT SCRQoL for users of social care services (the ASCOT) [30] in a study of older social care users in the United Kingdom [51]. The ASCOT-Carer and CES are both measures of carer care-related quality of life, albeit with differences in the measurement constructs (i.e. social care-related quality of life (ASCOT-Carer) and carer experience (CES)). By combining the CES and ASCOT-Carer items in the analysis presented here, we sought to determine whether the items could be reduced to the same underlying constructs. The EQ-5D-3 L items were also included in the EFA. It was expected that these items would not load onto the factor(s) as the ASCOT-Carer and/or CES, as the EQ-5D-3 L is a measure of a different construct that is distinct to care-related quality of life (i.e. health-related quality of life).

As the CES and EQ-5D-3 L items are scored on three-levels and the ASCOT-Carer on four-levels of response, the factor analysis was run on the correlations between variables. Since Pearson correlation coefficients can lead to incorrect conclusions with ordinal variables [52, 53], polychoric correlations were calculated and applied to the EFA. Bartlett’s test of sphericity [54] and the Kaiser-Meyer-Olkin (KMO) measure of sampling adequacy [55] were used to test whether EFA was appropriate. Mardia’s test for skewness was used to evaluate whether the data were multivariate normal [56]. A combination of visual inspection of the Scree plot, parallel analysis and consideration of the Kaiser criterion (eigenvalue> 1) was used to determine the number of factors [57]. Oblique oblimin rotation of the factors, which allows for correlated factors unlike orthogonal rotation, was applied to support the interpretation of the model [57]. Factor loadings of ≥0.40 are regarded as reliable for interpretation [58]; therefore, we only report factor loadings ≥0.40.

## Results

The characteristics of the sample are shown in Table 2. The majority of the sample were female (58.9%) and aged over 65 years (42.9%). Of those aged 64 or younger, the majority were aged 45–64 years (40.8% of the sample). This is comparable with the national estimate that 61% of carers in England are female and 42% are aged 45–64 years; however, the study sample has an older profile than the national estimate of 25% of carers aged over 65 years [59]. Correspondingly, the sample had a higher proportion of carers retired from paid employment (46.2%) compared with the estimate of the population of carers in England (27%) [59].

**Table 2:** Descriptive statistics of sample (*n* = 387)

	*N* (%) or (range), mean ± SD
Male	159 (41.1%)
Aged ≥65 years	166 (42.9%)
In paid employment	102 (26.4%)
Number of activities of daily living (ADLs) the care-recipient is unable to complete ^c^	(0 to 8), 3.39 ± 2.67
Carer and care-recipient live together	297 (76.7%)
Caregiving for ≥10 years	203 (52.5%)
Hours of caring ≥50 h per week ^c^	167 (43.2%)
*Quality of life measures* ^c d^	
ASCOT-Carer Index	(0 to 1), .72 ± .23
CES Index	(10.8 to 100), 68.70 ± 17.78
EQ-5D-3 L Index	(−.12 to 1), .76 ± .25
*Measures or items for construct validity analysis (hypothesis testing)*	
Overall quality of life ^d^	(1 to 7), 4.60 ± 1.04
Self-rated health (bad or very bad) ^a^	64 (16.5%)
UCLA 3-item loneliness scale ^d^	(3 to 9), 4.60 ± 1.94
Frequency of telephone contact with friends and family	(1 to 5), 4.23 ± .90
Frequency of face-to-face contact with friends and family	(1 to 5), 3.61 ± .97
Self-determination scale: choice subscale ^c d^	(1 to 5), 3.50 ± 1.17
Relationship rewards scale ^c d^	(1 to 12), 9.19 ± 2.64
Extremely, very or quite satisfied with social care services ^b c^	225 (58.1%)

^a^Base category: Rated as fair, good or very good

^b^Base category: Neither satisfied/dissatisfied, very or extremely dissatisfied with care

^c^Missing data:

Number of ADLs unable to complete alone or without help (n = 5)

Hours of caring per week (n = 1)

ASCOT-Carer (*n* = 3); CES (*n* = 8); EQ-5D-3 L (n = 3)

Self-determination scale (*n* = 1)

Relationship rewards scale (n = 3)

Satisfaction with social care service (*n* = 9)

^d^The full range for each measure is:

ASCOT-Carer Index, 0 (lowest) to 1 (highest) SCRQoL

CES, 0 (lowest) to 100 (highest) QoL

EQ-5D-3 L Index, − 0.594 (lowest) to 1 (highest) HRQoL

Overall quality of life, 1 (so bad it could not be worse) to 7 (so good it cannot be better)

UCLA 3-item loneliness scale, 3 (least) to 9 (most lonely)

Self-determination scale: choice subscale, 1 (least) to 5 (most choice/autonomy)

Relationship rewards scale, 0 (lowest) to 12 (highest relationship reward)

**Fig. 1:**
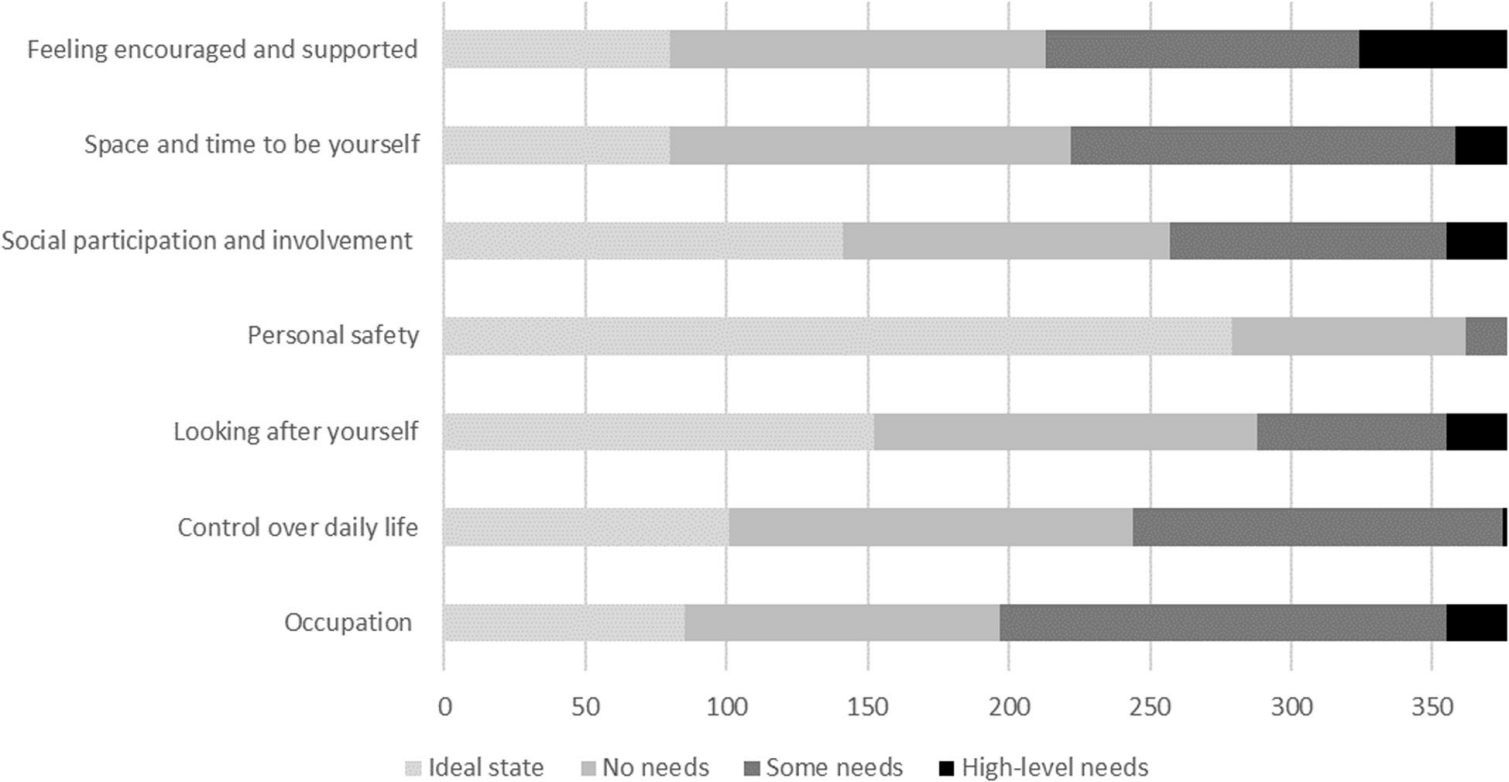
Distribution of responses to ASCOT-Carer items

**Fig. 2:**
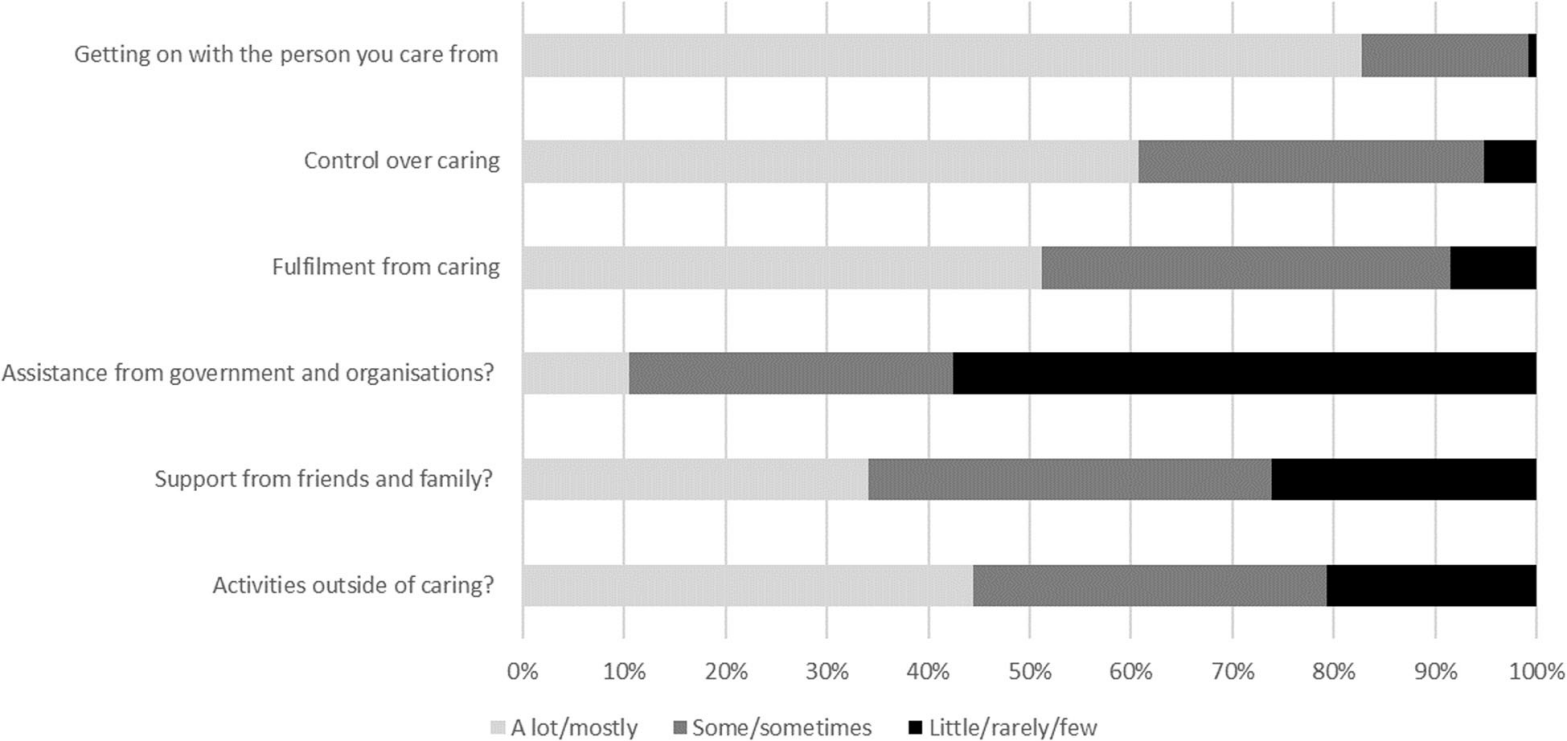
Distribution of responses to CES items

The distribution of responses to the items in the carer care-related quality of life measures, the ASCOT-Carer and CES, are shown in Figs. 1 and 2.

Pearson correlation coefficients are shown in Table 3. All hypothesised differences in correlations were observed, with two exceptions (see Table 4). These results provide evidence for the construct validity of the instruments as three measures of the distinct, but related, constructs. The EQ-5D-3 L is a measure of health-related quality of life. By contrast, the ASCOT-Carer and CES are measures of broader QoL, beyond health. The ASCOT-Carer captures aspects of carers’ quality of life that may be influenced by social care services and support (i.e. social care-related quality of life). The CES is a measure that captures carer experience more broadly.

**Table 3:** Pearson correlation coefficients

	ASCOT-Carer Index	CES Index	EQ-5D-3 L Index
CES Index	0.59**	–	0.36**
*CES: Activities outside caring*	0.63**	–	0.41**
*CES: Support from family and friends*	0.37**	–	0.14
*CES: Assistance from organisations and government*	0.14	–	0.14
*CES: Fulfilment from caring*	0.32**	–	0.15
*CES: Control over caring*	0.12	–	0.17
*CES: Getting on with the person you care for*	0.27**	–	0.15
EQ-5D-3 L Index	0.36**	0.36**	–
*EQ-5D: Mobility*	0.21**	0.18*	–
*EQ-5D: Self-care*	0.12	0.13	–
*EQ-5D: Usual activities*	0.19*	0.24**	–
*EQ-5D: Pain/discomfort*	0.24**	0.22**	–
*EQ-5D: Anxiety/depression*	0.40**	0.39**	–
Self-rated health (5-point scale)	0.47**	0.40**	0.61**
Overall quality of life (7-point scale)	0.61**	0.55**	0.44**
Self-determination scale, choice subscale	0.65**	0.51**	0.28**
Relationship rewards scale	0.38**	0.45**	0.17
UCLA 3-item loneliness scale	−0.57**	−0.50**	− 0.25**
Frequency of telephone contact with friends and family (5-point scale)	0.24**	0.26**	0.16
Frequency of face-to-face contact with friends and family (5-point scale)	0.31**	0.36**	0.10
Satisfaction with social care services (7-point scale)	0.38**	0.34**	0.12

*p < 0.05, **p < 0.01

**Table 4:** Hypothesised associations for construct validity of ASCOT-Carer, CES and EQ-5D-3 L

	Expected different observed?
ASCOT-Carer scores are 0.1 … than EQ-5D-3 L scores	
1.More strongly correlated to CES	Yes
2. Less strongly correlated to self-rated health	Yes
3. More strongly correlated to overall QoL	Yes
4. More strongly correlated to the SDS choice subscale	Yes
5. More strongly correlated to relationship rewards scale	Yes
6. More strongly correlated to UCLA loneliness subscale	Yes
7. More strongly correlated to frequency of telephone contact with friends and family	Yes
8. More strongly correlated to frequency of face-to-face contact with friends and family	Yes
9. More strongly correlated to satisfaction with social care services	Yes
ASCOT-Carer scores are 0.1 … than CES scores	
10. More strongly correlated to the SDS choice subscale	Yes
11. Less strongly correlated to relationship rewards	No
12. More strongly correlated to the UCLA loneliness subscale	No
ASCOT-Carer scores are within 0.1 the same correlation with … than CES scores	
13. Overall QoL	Yes
14. Self-rated health	Yes
15. Frequency of telephone contact with friends and family	Yes
16. Frequency of face-to-face contact with friends and family	Yes
17. Satisfaction with social care services	Yes

Two hypothesized differences were not observed related to the comparison of the ASCOT-Carer and CES (see Table 4). First, it was hypothesised that relationship rewards, or positive aspects of the relationship between the carer and care recipient, would more strongly associated to the CES than ASCOT-Carer. This is because one of the five CES domains captures how well the carer gets on with the care recipient (Table 4 (11)), whereas the ASCOT-Carer does not explicitly capture the carer/care-recipient relationship quality. As expected, the CES was more strongly correlated to relationship rewards than the ASCOT-Carer; however, this did not reach the 0.1 difference criterion (CES, *r* = .45; ASCOT-Carer, *r* = .38). Second, it was anticipated that the ASCOT-Carer would be more strongly correlated to UCLA loneliness score than the CES (Table 4 (12)) because the CES captures perceived social support, rather than social loneliness, whereas the ASCOT-Carer captures social relationships and the perception of social isolation. Again, the ASCOT-Carer was more strongly correlated to loneliness than the CES (CES, *r* = −.50; ASCOT-Carer, *r* = −.58), but the difference was less than the 0.1 difference criterion.

Table 5 presents the exploratory factor analysis of the ASCOT-Carer, CES and EQ-5D-3 L items. Bartlett’s test of sphericity indicated that the correlation between items is sufficient for a factor analysis to be conducted (Χ^2^(153) = 2300.32, *p* < 0.001). The KMO statistic of sampling adequacy was 0.88, which is well-above the minimum acceptable value of 0.50 for EFA to be applied [60]. Mardia’s test for skewness indicated that the data were not multivariate normal [56]; therefore, principal axis factoring was used for the factor extraction [57]. The Eigenvalues for the first four extracted factors were 6.51, 2.28, 1.18 and 0.68 respectively. This indicated a three-factor solution by the application of the Kaiser criterion (Eigenvalue< 1.00), which was also confirmed by visual inspection of the Scree plot for the point of inflection [57]. Parallel analysis using Horn’s test of factors [61] also indicated a three-factor solution.

**Table 5:** Oblimin-rotated factor loadings for the ASCOT-Carer, CES and EQ-5D-3 L items (*n* = 387)

Factor ^a^					
Measure	Item	1	2	3	Uniqueness
ASCOT-Carer	Occupation	0.86			0.29
	Control over daily life	0.87			0.24
	Self-care	0.66			0.43
	Personal safety	0.43			0.55
	Social participation	0.82			0.27
	Time and space to be yourself	0.84			0.31
	Feeling supported and encouraged	0.68			0.56
CES	Activities outside caring	0.76			0.32
	Support from friends and family	0.44			**0.79**
	Assistance from organisations /government				**0.97**
	Fulfilment from caring			0.50	**0.61**
	Control over caring			0.69	0.54
	Getting on with the person you care for			0.78	0.32
EQ-5D-3 L	Mobility		0.83		0.28
	Self-care		0.85		0.25
	Usual activities		0.92		0.17
	Pain		0.72		0.45
	Anxiety/depression				**0.67**
	Eigenvalue	6.51	2.28	1.18	
	Proportion of variance	59.3%	80.1%	90.1%	

^a^ We only present the highest factor loading per item, which is also > 0.40

Items with uniqueness > 0.60 are shown in bold text

The first factor included all seven of the ASCOT-Carer items and two of the CES items (activities outside caring, support from friends and family). These items all relate to the construct of social care-related quality of life (i.e. aspects of quality of life that may be influenced by social care support). All of these items had low uniqueness (≤0.60) with the exception of CES *support from friends and family*. Together these items explained 59.3% of the variance in the items. The second factor comprised the EQ-5D-3 L items, except for anxiety/depression, and accounted for 10.0% of the variance. These items relate to physical aspects of health-related quality of life. The third factor comprised three of the six CES items: *fulfilment from caring, control over caring* and *getting on with the person you care for*. These relate to the carer experience (i.e. the subjective experience of caring from the perspective of the carer). This second factor accounted for 20.8% of the variance. Only one of these three items had high uniqueness (*fulfilment from caring*). Two items did not show factor loadings of greater than the threshold for reliability set at 0.40 [58]. These were the CES item *support from government and organisations* (Factor 1 = .18; Factor 2 = .03; Factor 3 = −.0.2) and the EQ-5D-3 L item *anxiety / depression* (Factor 1 = .36; Factor 2 = .19, Factor 3 = .22), both of which also had high uniqueness (≥0.60).

## Discussion

This study tested the construct validity of the ASCOT-Carer, CES and EQ-5D-3 L index scores and the dimensionality of the items using data collected from a survey of carers in England. The findings of hypothesis testing broadly support that the three measures capture different constructs. Specifically, the EQ-5D-3 L is a measure of carer outcomes in terms of the carer’s health, whereas the CES and ASCOT-Carer measures capture broader aspects of QoL ‘beyond health’. This is important because it is known that the experience of caregiving may affect these broader aspects of QoL, like the ability to stay in work, or social relationships. Correspondingly, social care interventions via services or policy are often designed to address these broader aspects of quality of life, rather than health (e.g. support to stay in employment) [6–9]. Therefore, in order to ensure that the effects of health and social care interventions are adequately captured, it is important to use outcome measures that are able to capture these broader aspects of QoL. These findings add to earlier studies of the construct validity of the CES and ASCOT-Carer measures [25, 29] and also directly compares these measures with each other, as well as with the EQ-5D-3 L, as a measure of health-related QoL.

While the CES and ASCOT-Carer are both measures of carers’ QoL ‘beyond health’ that are distinct from the concept of health-related QoL measured by the EQ-5D-3 L, the construct validity analysis provides evidence that the CES and ASCOT-Carer also capture distinct constructs. The ASCOT-Carer captures aspects of QoL that are typically targeted by social care services in England (i.e. social care related quality of life) and was more closely related to carers’ perceived satisfaction with social care support than the CES. By contrast, the CES captures more general aspects of carers’ experience, including caring-related aspects of QoL that may be beyond the scope of social care interventions.

In the construct validity analysis, however, two of the hypothesised differences in correlations were not observed. It had been expected that the CES would be more strongly related to relationship reward (quality) between the carer and care-recipient than the ASCOT-Carer because the CES includes an item that seeks to capture relational quality (*getting on with the person you care for*). The ASCOT-Carer was anticipated to be more strongly related to loneliness, due to the related concept of ‘feeling isolated’ captured by the *social participation and involvement* item. However, the observed differences in correlation were less than the criterion applied for this study. This indicates an area of potential overlap between the two measures. Further research could provide evidence of the conceptual overlaps between these items around the dimensions of relationships, social participation and loneliness (e.g. through in-depth qualitative interviews to explore these items).

In the analysis of the factor structure of the ASCOT-Carer, CES and EQ-5D-3 L items combined, it was found that the CES and ASCOT-Carer items partially measure the same construct, whereas the EQ-5D-3 L items load onto a separate factor. With regard to the ASCOT-Carer and CES overlap, the CES items of *activities outside of caring* and *social support* were found to load onto the same factor as the seven ASCOT-Carer items. These two attributes have some conceptual overlap with the ASCOT-Carer attributes of *Occupation - doing things I value and enjoy* and *Social contact and participation,* however, the CES items relate more specifically to caregiving experience. For example, ASCOT-Carer *Social contact and participation* captures the broad outcome state of an individual’s satisfaction with the quality and quantity of social contact and connectedness. By contrast, the CES social support item refers more narrowly to the level of social support from friends and family with respect to caregiving, yet still relates to social contact.

Three of the six CES items loaded onto a separate factor to the ASCOT-Carer. The attributes of *fulfilment from caring*, *control over caring*, and *getting on with the care-recipient* may be conceptualised as aspects of the caregiving experience [27], which are distinct from the construct of aspects of QoL that may be affected by social care interventions. For example, *Getting on with the care-recipient* may be indirectly and weakly influenced by social care services, however, the quality of the existing relationship and other factors are likely to be more important [26]. Likewise, the carer’s experience of *fulfilment from caring* is not a concept that carers are able to relate to the effect of social care services [26]. Unlike control over daily life, which may be affected by social care support [26], *control over caring* is the carers’ experience of their ability to influence the care provided by health, social care and other local services (see Table 1). The CES item that captures the *amount* of assistance from organisations and the government does not load onto either of the two factors identified in the analysis.

Four of the five EQ-5D-3 L items loaded onto a separate factor with no overlap with the ASCOT-Carer or CES. One of the five items (anxiety/depression) did not load onto any of the factors, with the minimum loading criterion of >.40. Of the three factors, this item had the highest loading for factor one, along with the seven ASCOT-Carer items and two CES items. Therefore, study provides evidence that the ASCOT-Carer, like the service user version of ASCOT [47], and also the CES, only captures physical health in a limited way; however, there may be more overlap with psychological health. This finding is consistent with the conceptual basis of the CES and ASCOT-Carer as measures of carers’ outcomes, beyond health.

The limitations of the study should be considered when interpreting the results. This paper presents analysis based on a sample of carers in England. Data was collected from a heterogeneous sample of carers identified through users of publicly-funded social care services. Due to the study methodology, the sample is not representative of carers in England. While some of the sample characteristics are consistent with population estimates [59], older adults are overrepresented. Even with this limitation, the study provides further evidence of the validity of the CES and ASCOT-Carer with a heterogeneous sample of carers that is consistent with other studies [25, 29] and how these measures compare to the EQ-5D-3 L. Further work is needed to establish the validity and comparability of the measures in other countries with different social policy, cultural and linguistic contexts.

In summary, this study provides evidence of construct and structural validity of the ASCOT-Carer, CES and EQ-5D-3 L as carer outcome measures. The items from these measures load onto three separate factors. These relate to (1) *social care related quality of life* (i.e. aspects of broader quality of life that may be affected by social care services); (2) *carer experience* related to the *process* of caregiving, which are outside the direct influence of social care services; and (3) physical *health-related quality of life*. The strong correlation between the ASCOT-Carer/CES suggests that (economic) evaluation studies could use one or other of these two measures, rather than both together. There is some evidence that the ASCOT-Carer may be better suited to the evaluation of social care interventions, however, further research would usefully inform an understanding of the conditions under which these two measures perform best. The EQ-5D-3 L captures the distinct construct of health-related quality of life, so may be used alongside the ASCOT-Carer or CES to consider both health-related and ‘beyond health’ outcomes. Further work is required to establish whether this approach may potentially double-count health effects, especially with regard to psychological health.

## Conclusions

The results of this study indicate that the ASCOT-Carer and CES are promising measures for the evaluation of health and social care services for carers that capture aspects of quality of life ‘beyond health’. The choice of whether to use the ASCOT-Carer or CES depends on the study objectives. If the study seeks to evaluate social care services, defined as services ‘for’ the care-recipient and/or the carer, the ASCOT-Carer may be more suitable. If the study aims to measure carer experience more broadly, the CES may be more suitable. Further research is needed to establish the conditions under which each measure performs best, to inform the appropriate use of these measures in evaluation studies of health and social care interventions or policy.

## Data Availability

The raw data from the study reported in this article is not freely available because we do not have consent for publication of these data.

## Abbreviations

ADLs: Activities of daily living
ASCOT: Adult Social care outcomes toolkit
ASCOT-Carer: Adult social care outcomes toolkit for carers
CES: Carer experience scale
EFA: Exploratory factor analysis
EQ-5D: EuroQol five-dimension questionnaire
EQ-5D-3 L: EuroQoL five-dimension questionnaire with three levels of response
HRQoL: Health-related quality of life
ICECAP-O: ICEpop CAPability measure for Older people
INT4: Interview with four levels of response
KMO: Kaiser-Meyer-Olkin
QALY: Quality-adjusted life years
QoL: Quality of life
SCRQoL: Social care-related quality of life
SDS: Self-determination scale
